# Le syndrome des enfants battus: aspects cliniques et radiologiques

**DOI:** 10.11604/pamj.2016.24.68.8543

**Published:** 2016-05-20

**Authors:** Zied Jlalia, Talel Znaigui, Mahmoud Smida

**Affiliations:** 1 Service d'Orthopédie Infantile- Institut MT Kassab, Tunisie; 2 Hôpital Militaire de Tunis, Tunisie

**Keywords:** Fracture, maltraitance, enfant, neuroradiologie, Fracture, abuse, child, neuroradiology

## Abstract

La maltraitance physique des enfants ou le syndrome des enfants battus est responsable de plus de 75.000 décès par an en France. Ce problème de santé publique reste sous diagnostiqué en Tunisie et dans le monde. Le chemin a été laborieux pour la reconnaissance du syndrome des enfants battus dans certaines sociétés même occidentales. Nous avons voulus exposer ce problème aux praticiens afin qu'il soit mieux diagnostiqué et pris en charge. La maltraitance physique des enfants est appelée à tort syndrome de Silverman qui ne regroupe en fait que les lésions squelettiques chez ces enfants tels que les fractures. Mots clés: Fracture, maltraitance, enfant, neuro-radiologie

## Abstract (en)

Physical child abuse or battered child syndrome is responsible for over 75.000 deaths per year in France. This public health problem is under-diagnosed in Tunisia and in the world. The path toward the recognition of battered child syndrom was arduous even in some western societies. This study aims to highlight this problem to healthcare practitioners in order that accurate diagnosis and appropriate management may be provided. Physical child abuse has wrongly been referred to as Silverman syndrome that includes only skeletal lesions in children such as fractures.

## Introduction

Le syndrome de maltraitance de l'enfant regroupe, la maltraitance physique, psychologique, sexuelle et sociale infligé à l'enfant par une tierce personne. C'est un grave problème de société et de santé publique, 40 millions d'enfants sont touchés dans le monde [1]. La maltraitance de l'enfant était pratiquée par les anciennes civilisations, comme c'est le cas de la célèbre cité de sparte ou les enfants handicapés étaient jetés dans le ravin du Taygète [1]. Autrefois on parlait de cruauté, brutalité ou correction physique sévère infligés à l'enfant, le chemin vers la reconnaissance de cette maltraitance fut long, résultat d'une collaboration entre plusieurs disciplines. Ce problème touche les enfants de tous les âges et de tous les milieux. Le médecin doit être sensibilisé à ce problème pour mieux le diagnostiquer devant une symptomatologie souvent diverse et peu spécifique.

## Patient et observation

**Figure 1:**
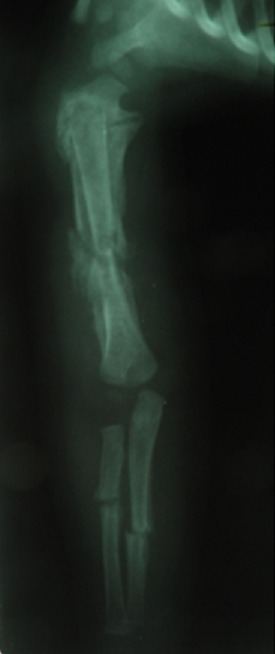
Réaction périosté, humérus droit

**Figure 2:**
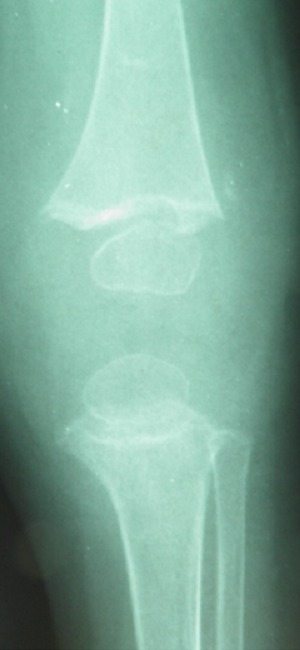
Arrachement osseux métaphysaire de l'extrémité inférieure du fémur

**Figure 3:**
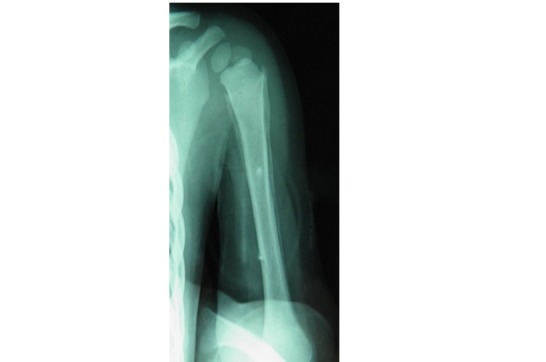
Réaction périosté, humérus droit

**Observation 1:** Il s'agit d'une fille âgée de 45 jours, 1 er enfant d'un jeune couple mère au foyer et père récemment devenu chômeur. Admise aux urgences pour une impotence fonctionnelle totale du membre supérieur droit. Les parents ne rapportent pas la notion de traumatisme récent mais évoquent un traumatisme obstétrical du membre supérieur droit traité orthopédiquement. Le tiers inférieur du bras droit était tuméfié, la palpation du bras droit était douloureuse déclenchant la crispation et les pleurs du nourrisson. La palpation des 2 fémurs était aussi douloureuse avec choc rotulien au niveau des 2 genoux. La mère signale que son enfant ne s'alimente plus normalement, par ailleurs le nourrisson était apyrétique mais avec une biologie perturbé en faveur d'un syndrome inflammatoire. Aucune prise médicamenteuse n'a était rapporté. Les radiographies standard ont trouvé de multiples réactions périostés intéressant l'humérus droit ( Figure 1), les 2 fémurs (Figure 2) et les 2 tibias (Figure 3). Ces réactions périostés étaient sur toute la diaphyse de ces segments osseux avec des fractures décollements épiphysaires stade II selon Salter et Harris au niveau des métaphyses tibiales supérieures. L’échographie a trouvé des hématomes sous périosté pandiaphysaire ainsi qu'un épanchement articulaire au niveau des 2 coudes et du genou gauche. Le diagnostic d'ostéomyélite aigue multifocale a été évoqué et une antibiothérapie a été démarrée. Néanmoins l'absence de circonstance claire de cette impotence fonctionnelle, l'hémoculture négative, la présence de décollements épiphysaires des 2 tibias et surtout la bonne évolution d'emblée font redresser le diagnostic. Ainsi une analyse échographique plus fine a montré que les décollements périosté étaient en rapport avec des hématomes sous périostés survenus très probablement après des mouvements de torsion des membres. Les Parents jeunes et inexpérimentés avec conditions socioéconomiques difficiles, le jeune âge du patient, la nature et le nombre des lésions squelettiques le tout plaidait en faveur d'un syndrome de Silverman.

**Figure 4:**
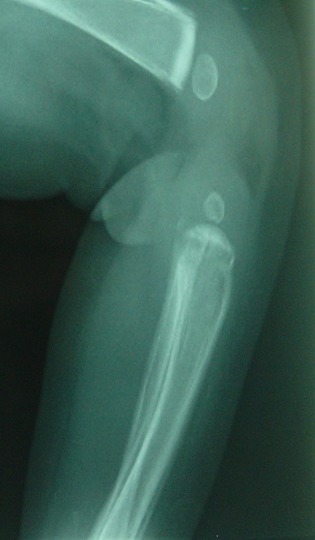
DESII des métaphyses tibiales supérieures et réaction périosté pandiaphysaire

**Observation 2:** Un garçon de 10 mois, sans antécédents pathologique notables admis par le biais des urgences pour une impotence fonctionnelle totale du membre supérieur droit à la suite d'un accident domestique selon la mère, qui rapporte aussi la notion d'accidents domestiques survenus il y a 20 jours, ayant entraîné une fracture des 2 os de l'avant-bras droit. Le nourrisson était fébrile avec une rhinorrhée et sibilants au niveau des 2 champs pulmonaires a l'auscultation. La radiographie standard a trouvé des fractures d’âge différent de l'humérus ainsi que des 2 os de l'avant-bras droit (Figure 4), ainsi qu'un hématome sous périosté diaphysaire de l'humérus droit. Le diagnostic d'ostéomyélite aiguë( OMA) de l'humérus droit a été évoqué, devant le décollement périosté et l’état fébrile du nourrisson. Une fracture pathologique de l'humérus droit a été aussi évoqué, les 2 diagnostiques ont étaient abandonnés. La mère a reconnu avoir fait subir à son enfant des mouvements de torsion de l'humérus droit. Bonne évolution après traitement de sa virose avec consolidation des fractures à long terme.

## Discussion

Il est difficile de proposer une définition à la maltraitance de l'enfant car elle est variable en fonction des cultures et de l’évolution des connaissances de la société. L'enfant mal traité est celui qui est victime de violences physiques, psychologiques, sexuelles ou de négligences ayant des conséquences graves sur son développement physique et/ou psychologique [2]. Jadis les enfants étaient mutilés pour en faire des mendiants plus rentables, ou battus par des parents sadiques. Même les princes n’étaient pas épargnés, en effet Henri IV roi de France ordonna à sa gouvernante de fouetter son fils, le futur roi Louis XIII [3]. Plusieurs médecins se sont intéressés à ce problème mais ils se limitaient à des constatations sans véritable analyse ou étude. Le premier médecin qui a décrit d'une façon systématique les manifestations cliniques, les circonstances et les conséquences envers les enfants d'une maltraitance fut Auguste Ambroise Tardieu. Tardieu médecin légiste Français s'est intéressé à tous les aspects de la maltraitance de l'enfant, en 1860 il a publié un article basé sur l'analyse de 32 observations de violences physiques, dont 18 parmi elles étaient décédés [3]. Malgré sa notoriété Tardieu échoua dans sa tentative d’éveiller l'attention de ses contemporains sur cette réalité, en effet à son époque l'autorité paternelle était un droit inviolable. Aux Etats unis le premier mouvement de protection des enfants à vue le jour après l'histoire de Mary Ellen Wilson. Cette fillette de 11 ans placé en famille d'accueil avait subi une maltraitance physique et morale par ses parents adoptifs, alerté par une voisine de la famille un groupe d'avocats New-yorkais se sont saisis de l'affaire et ont fondé le premier organisme Américain pour la protection des enfants [3]. En France les premières lois ont vu le jour en 1889. Au début du 20^eme^siècle l'idée que des parents puissent maltraiter leurs enfants fut difficile à accepter par les médecins. John Caffey radiologue pédiatre américain s'intéressa à la présence d'une réaction périostée chez certains nourrissons, il évoque l'intoxication à la vitamine A, l'hyperostose corticale infantile et enfin la violence physique entraînant des lésions squelettiques particulières et propre a l'enfant [3]. Caffey avait constaté l'association d'hématomes sous-duraux à des fractures d'arcs postérieurs de côtes, ce qui définira plus tard le syndrome du nourrisson secoué. L'assistant de John Caffey, Frédedric Noah Silverman a continué le travail de son mentor en introduisant à la médecine moderne la notion de maltraitance physique des enfants, avec lésions squelettiques particulières et la responsabilité pénale des parents. Un autre pionnier fut Henry Kempe, directeur du département de pédiatrie au Colorado [3]. Kempe a vu afflué dans son service plusieurs cas de fractures et de lésions cutanés chez des nourrissons et des enfants dans un contexte douteux, avec des parents et des enfants à profils particuliers. Il organisa une plénière au congrès de “l'American Academy of pediatrics" avec comme titre “the battered-child syndrome". En 1957 Kempe et Silverman ont conjugué leurs efforts dans un article qui a regroupé 302 observations d'enfants battus, ce travail avait signé l'acte de naissance du syndrome de Silverman.

### Quand penser à la maltraitance?

Le diagnostic de maltraitance physique n'est pas toujours facile, en effet la limite entre maltraitance et punition parentale sévère et mal définie. Aucune lésion n'est pathognomonique, la peau et l'os sont les plus touché [4]. La règle en médecine pédiatrique est de se fier à la parole des parents mais ces derniers mentent souvent sur les circonstances de survenue des lésions. Les enfants maltraités parlent peu et peuvent parfois mentir par craintes des abuseurs, les témoins sont rares car ces maltraitances surviennent souvent dans un contexte familial. La loi Française considère toute punition physique causant un traumatisme allant au-delà d'une marque transitoire, celle infligé à un enfant de moins de 2 ans, celle par un objet contendant, celle comportant des coups sur la tête, comme une maltraitance physique [1]. Le diagnostic des enfants battus doit être présent à l'esprit de l'orthopédiste pédiatre lors de l'examen de chaque enfant qui consulte pour un traumatisme. En effet l'hypervigilance, la peur, la passivité, l'agressivité, et l'aspect déprimé de ces enfants doivent attirer l'attention du praticien [2]. La maltraitance peut se voir à tout les âges mais ces enfants ont souvent moins de 3 ans [5]. Lorsque les blessures de l'enfant ne sont pas graves la consultation et tardive et se fait par des personnes autre que les parents, souvent une grand-mère ou une tante. L'enfant maltraité présente souvent des signes de négligence, il présente une dermatite de couche, un retard pondéral et de multiples blessures de différents âges. L'atteinte de différents systèmes: peau, os, muscles et système nerveux central est en faveur de maltraitance physique.

### Facteurs de risques et étiologies de la maltraitance des enfants

Plusieurs études ont été faites dans le but de dresser le profil du parent maltraitant et de l'enfant maltraité, ces études ont servi à déterminer certains facteurs de risques mais sans véritable profil particulier pour l'abuseur ou pour les enfants maltraités. Une grossesse mal désirée, une naissance prématurée, un enfant porteur d'handicap et/ou une maladie chronique semblent précipiter la maltraitance des parents pour leurs enfants. Les parents jeunes, impulsifs, pauvres, bas quotient intellectuel et addiction à l'alcool et/ou aux drogues représentent des facteurs de risques de maltraitance [3]. Les pleurs excessifs, les réveils nocturnes, les troubles de l'alimentation de l'enfant, la désobéissance et le conflit conjugal représentent autant de facteurs déclencheurs de maltraitance physique des enfants.

### L'examen clinique dans le syndrome des enfants battus

L'examen se fera dans des conditions optimales pour le médecin et pour l'enfant et sa famille. On commencera par préciser les circonstances de survenu des lésions chez l'enfant. Habituellement l'histoire est prise auprès des parents qui accompagnent l'enfant, si l'enfant est assez âgé pour parler il serait mieux de lui poser directement les questions. Il faut mettre l'enfant en confiance, éviter les suggestions de faits mais lui demander simplement de dire sa version des événements et si l'enfant refuse de parler, il ne faut pas insister [3]. Souvent lorsque un enfant raconte avoir été victime d'agression, il dit la vérité. L'enfant peut aussi mentir et nier la maltraitance afin de protéger son agresseur, par soucis de ne pas être au centre d'un problème ou par peur de causer du tort à un parent. Il est important de préciser la date, l'heure et la présence ou non de témoins. Par exemple en cas de chute il faut préciser la hauteur, la position de l'enfant ainsi que la nature de la surface de réception. Il faut connaitre la situation socio-familiale, les antécédents familiaux (Problèmes métaboliques, troubles de la coagulation, fractures, maladies chroniques). Afin de faciliter le contact il faut s'ajuster au niveau du développement de l'enfant et connaitre son vocabulaire, il est aussi préférable que le questionnaire se fasse hors la présence des parents. Pour le nourrisson on s'intéressera à l'histoire de la grossesse et de l'accouchement, en effet la prématurité est un facteur déclencheur de maltraitance surtout par la mère. Se méfier lorsque l'histoire change d'un questionnaire à l'autre chez une même personne et lorsque il y a une discordance entre l'histoire et le développement de l'enfant, ou une discordance entre les blessures et l'histoire rapporté par les parents [3]. Après avoir précisé les circonstances de survenue une évaluation de l’état général est nécessaire, on trouve des enfants craintifs, repliés sur eux même, avec retard pondéral, signes de négligences vestimentaires et corporelles. L'examen de la peau est très important, il est fait chez un enfant nu, chaque lésions doit être située et photographiée. L'examen des phanères peu aussi révéler des signes de maltraitance, des alopécies traumatique et des ecchymoses péri-unguéale. A l'examen musculo-squelettique on cherchera des douleurs et des déformations osseuses. Lorsque l'abdomen est sensible, distendu où a paroi ecchymotique, une lésion viscérale est suspectée [5]. En cas de suspicion de maltraitance physique d'un enfant une hospitalisation permettra une protection de l'enfant en attendant de préciser le diagnostic. L'orthopédiste pédiatre doit documenter par des photos tout ce qu'il constate comme lésions chez l'enfant.

### La place des examens complémentaires

Les examens complémentaires ont pour objectifs de rechercher des étiologies aux lésions constatées et de faire un bilan lésionnel précis. Une numération de la formule sanguine et un bilan d'hémostase sont nécessaires en cas de saignement ou d'ecchymoses. Lorsque l'enfant est âgé de moins de 2 ans et en cas de forte suspicion de maltraitances physiques, “l'American collège of radiology” recommande de faire systématiquement les radiographies du crâne, thorax, bassin, et rachis. Des radiographies de l'humérus, des 2 os de l'avant-bras, du fémur, et du tibia seront aussi réalisés et chercheront des fractures anciennes ou passées inaperçues à l'examen [6]. Si le bilan radiologique fait en urgence revient normal, on refait un autre bilan radiologique après 15 jours à la recherche de réaction périostée. Chez les nourrissons et les jeunes enfants les trajets veineux peuvent être pris pour des fractures ils ne doivent pas égarer le diagnostic. Dans des cas extrêmes une scintigraphie osseuse est réalisée, elle est assez sensible en cas de fracture de cote et de réaction persistée précoce [6]. La tomodensitométrie de l'os est difficile d'interprétation chez le nourrisson et le jeune enfant car l'os est très riche en eau à cet âge, en effet cet examen permet de mesurer la densité minérale et ne permet pas l’évaluation de la résistance de l'os. La tomodensitométrie cérébrale est très utile en cas de traumatisme crânien ou lorsqu’ un syndrome du “bébé secoué” est suspecté. L'imagerie par résonance magnétique est d'utilisation fréquente surtout en cas de traumatismes crâniens, et lorsque le scanner n'apporte pas d'explication après une altération de l’état de conscience ou en cas de crises convulsives chez les nourrissons [7].

### Les signes cutanés de la maltraitance physique chez l'enfant

Ce sont généralement des blessures mineures, elles sont constatées par l'orthopédiste pédiatre lors de la consultation pour un traumatisme ou pour une autre cause. Elles siègent volontiers au niveau des joues, cou, thorax, abdomen, fesses, région lombaire, organes génitaux, face interne des cuisses. Toute ecchymose chez un nourrisson avant l’âge de 6 mois nécessite une explication, en effet les ecchymoses sont rares avant l’âge de la marche [5]. Les seules lésions “normales” a cet âge sont les éraflures au vissage auto-infligés. Certains objets utilisés pour faire mal à l'enfant comme une ceinture, une baguette ou un objet brûlant laissent une trace particulière. Lorsque un adulte serre le bras d'un enfant la force appliqué est constante est prolongée produisant un bris des capillaires au site de pression ceci aboutit a des ecchymoses épousant la forme des doigts. Lors d'une gifle la force appliqué est rapide mais intense le sang est chassé des capillaires, ceci provoque une hyper pression et l’éclatement de ces derniers, cliniquement on retrouve 5 fines lignes pétéchiales parallèles [3]. Les pincées produisent 2 ecchymoses rapprochées séparé par un espace de peau saine. Les morsures humaines causent des ecchymoses de forme incurvée, une distance supérieure à 3 cm entre les 2 canines est celle d'une mâchoire adulte [5]. Des cratères de 8 à 10 mm en rapport avec des brûlures de cigarettes peuvent être présents [3]. Les brûlures par immersion au niveau des membres en gants ou en bottes bilatérales et symétrique sont fréquentes et doivent toujours faire suspecter une maltraitance physique, en effet pour causer une brûlure du troisième degré chez un enfant avec une eau chauffée à 60 ° il faut au moins 5 secondes d'exposition, une durée difficile a supporter volontairement [4]. Les pertes de cheveux localisé et non uniforme suggèrent une alopécie traumatique. Les lésions cutanées sont suspectes par leur nombre mais aussi par l'existence de lésions d’âges différents. Néanmoins il existe quelques diagnostics différentiels, comme l'hémangiome, une vascularite, un syndrome d'Ehlers Danlos, qui s'accompagne d'une fragilité cutané ainsi que des malformations squelettiques [3], des lésions cutanées généralisées en rapport avec une intoxication médicamenteuse, et bien sur l’éventualité d'un traumatisme accidentel.

### Les lésions osseuses dans la maltraitance physique chez l'enfant

**Figure 5:**
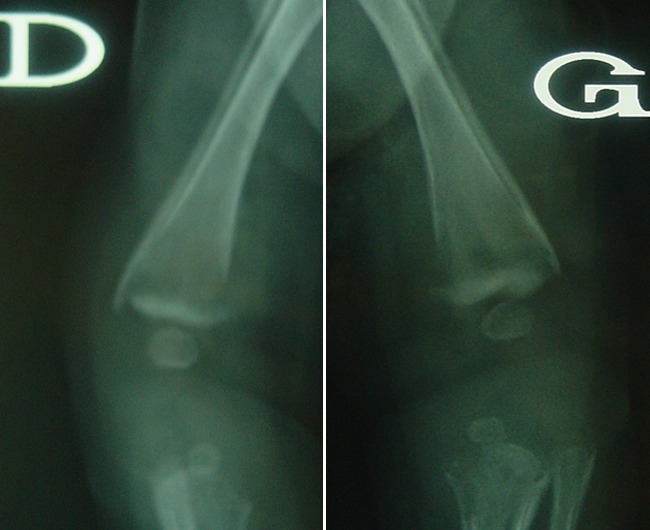
Réaction périosté diaphysaire des 2 fémurs

Les fractures occupent la 2^éme^ position dans la clinique de la maltraitance chez l'enfant. Mais toutes les fractures chez un enfant actif ne sont pas suspectes, quelques aspects son propre aux fractures survenant après maltraitance. Plus l'enfant est jeune plus la fracture est suspecte surtout si il est non ambulant. Le nombre de ces fractures doit attirer l'attention, habituellement on retrouve plusieurs fractures à âges radiologiques différents. Avant l’âge de 5 ans ces fractures sont localisées au niveau du crâne, clavicule, côte, humérus, sternum, fémur, après l’âge de 5 ans elles se localisent volontiers au niveau du tibia et à l'avant-bras. Chez le nourrisson on retrouve souvent des fractures métaphysaires souvent localisées au niveau du fémur distal, tibia et humérus proximal. Ces fractures ont été décrites pour la première fois par John Caffey, elles correspondent à de petits arrachements osseux des coins de la métaphyse(corner fracture)secondaire a une force de cisaillement lors des gestes de traction, de torsion ou d'accélération-décélération (Figure 5). Le trait de fracture est souvent parallèle à la physe et lorsqu'il parcourt toute la largeur de la métaphyse il réalise une fracture en anse de seau (bucket handle fracture). Les fractures de côtes du nourrisson sont toujours suspectes, la fracture de la première cote témoigne de la violence du traumatisme et à cet âge elle est très suggestive d'une maltraitance [6]. La fracture des cotes après accouchement dystocique et par voie basse est exceptionnelle vu la malléabilité de la cage thoracique du nouveau née, l’étude de 155 cas de ces accouchements n'a trouvé aucune fractures de cotes [6]. De même pour le massage cardiaque du nourrisson, en effet l’étude post mortem de 91 cas ayant subi une réanimation cardio-respiratoire ne trouve aucune fracture de cotes [6]. Le mécanisme de fractures de cotes chez les nourrissons maltraités serait donc particulier, il est le résultat de la conjugaison des efforts de 2 mains. L'agresseur saisis le thorax en plaçant le pouce en avant, la paume de la main sur le côté et les doigts en arrière de part et d'autres des apophyses épineuses du rachis, et secoue le nourrisson en comprimant le thorax qui est à cet age malléable. Les doigts compriment en arrière les apophyses transverses des vertèbres qui viennent s'appuyer sur les côtes et jouent ainsi le rôle d'un levier. Ceci entraîne des fractures bilatérales étagées de la partie postérieure des côtes, ainsi que des avulsions des apophyses épineuses [3, 6]. Ces lésions sont souvent associés à des hématomes sous duraux cette association constitue le syndrome du “bébé secoué” décrit par John Caffey en 1964 [8]. La fracture de cotes accidentelle est souvent unique et se voit après l’âge de 2 ans. Les fractures du crâne sont suggestives aussi de maltraitance elles touchent souvent l'os pariétale et sont multiples. Les fractures diaphysaires siègent le plus souvent sur l'hémicorps gauche car les enfants sont souvent appréhendés par un adulte droitier [3].

**Figure 6:**
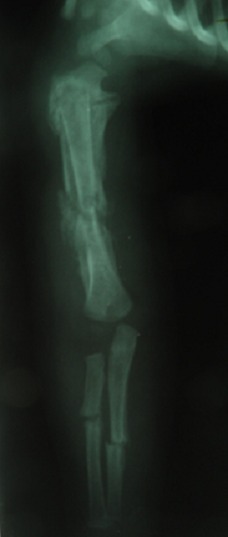
Réaction périsoté, suite à un hématome décollant le périoste de l'humérus

Une revue systématique de la littérature avait montré que 3 à 8% des lésions du rachis chez les enfants étaient dus à la maltraitance physique [9]. Le rachis de l'enfant est plus souple que celui de l'adulte par prédominance des structures cartilagineuses et une plus grande élasticité des ligaments, néanmoins des lésions vertébro-medulaire graves peuvent se voir. Les parents rapportent des histoires de traumatismes bénins (chute du lit, d'un déambulateur pour enfant), c'est ce qui attire l'attention de l'orthopédiste pédiatre. Les lésions du rachis cervical se voient avant l’âge de 2 ans. En effet la disproportion entre la tête et le reste du corps, une musculature du cou peu développées à cet âge et une orientation plus horizontale, que chez l'adulte des surfaces articulaires des vertèbres cervicales facilitent ces lésions [9]. Les vertèbres les plus touchées chez le nourrisson sont C1, C2 par contre les lésions du rachis cervical inférieur sont plus vu à partir de l’âge de 7-8 ans [9]. Chez les nourrissons les lésions du rachis cervical se voient souvent dans le cadre du syndrome du “bébé secoué”, elles sont accompagné de trouble de la conscience et de détresse respiratoire, des ecchymoses au niveau du cou dus a des mouvements de torsion sont parfois présentes. Ces lésions se voient beaucoup chez les patients atteints d'hypotonie musculaire, comme c'est le cas pour certaines formes d'insuffisance motrice cérébrale (IMC) qui est vécu comme un échec surtout par la mère [9]. A chaque fois qu'une maltraitance physique est suspecté chez un nourrisson la radiographie du rachis cervical est systématique, on peut y voir une fracture corporelle d'une vertèbre, des arrachements étagés des apophyses épineuses, et un antélisthésis C2, C3 qui est normal jusqu'a l’âge de 7-8 ans. La radiographie du rachis cervicale peu être normale. Dans le cadre du “syndrome du bébé secoué” et en cas de tétraplégie avec radiographie normale un complément d'imagerie par résonance magnétique (IRM) cérébro-médullaire est systématique. En effet chez 18 nourrissons présentant des hématomes sous duraux, 8 cas présentaient des lésions médullaires du rachis cervical. Cet examen peu mettre en évidence un hématome sous dural et surtout un œdème médullaire hémorragique et compressif expliquant les déficits neurologiques [9]. La moyenne d’âge des lésions du rachis thoraco-lombaire est de 13.5 mois [9]. Cliniquement il y a une déformation du rachis, avec apparition parfois d'une cyphose lombaire, les déficits neurologiques sont fréquents surtout par lésion médullaire isolé qui est particulière à l'enfant et qui a été décrite dans le cadre du syndrome de Sciwora(spinal cord injury without radiographic abnormality in children). On estime que 25 à 30% des traumatismes crâniens chez les enfants de moins de 2 ans surviennent dans le cadre de la maltraitance physique [10]. Pour Ichord et al, la bilatéralité des hématomes sous duraux chez un nourrisson est très suggestive d'une maltraitance en effet sur 31 cas de syndrome du “bébé secoué”, 26 cas avaient présenté un hématome sous-dural (HSD) bilatéral, par contre pour 29 cas de traumatisme crâniens en dehors de toute maltraitance 20 nourrissons avaient présenté un HSD bilatéral [10]. Pour Wells l'association d'une bilatéralité de l'HSD, une hémorragie cérébrale inter hémisphérique, la présence de lésions ischémiques cérébrales d'origine hypoxique (atteinte ventilatoire par fracture de cotes ou lésion du tronc cérébral), une hémorragie rétinienne, et l'absence d'une fracture du crâne, cette association peut se voir dans le syndrome du"bébé secoué", sa sensibilité est de 84% et sa spécificité est de 83% ( 95% IC 74 - 89%) pour le diagnostic d'un syndrome de Silverman [10]. Par ailleurs et en dehors du syndrome de silverman l'HSD néonatal peut se voir en cas d'accouchement par forceps, dystocie et macrocéphalie du nouveau née [10]. Certaines maladies peuvent poser un diagnostic différentiel, comme la lymphohistiocytose hémophagocytaire et l'acidurie glutarique type I, cette dernière peut s'accompagner d'un saignement sous arachnoidien et d'une atrophie cérébrale [10]. La torsion du membre peut provoquer une réaction périosté ou un épaississement du périoste qui est a cet âge très vascularisé et peu adhérent à l'os, cette torsion provoque un saignement sous périosté qui s'organise en hématome (Figure 6). Dans un contexte fébrile et avec une biologie perturbée, ces véritables décollements périosté peuvent égarer le diagnostic [3]. La douleur et la fièvre avec un décollement périosté à l’échographie nous font penser à l'ostéomyélite aiguë. Des tumeurs osseuses peuvent être aussi évoquées, par ailleurs des réactions périostés de moins de 2 millimètres sont normales chez des nourrissons de moins de 6 mois, l'approche clinique permettra de faire la part entre maltraitance physique ou autres diagnostiques [3]. Ces fractures par leurs sièges et leurs fréquences doivent motiver la recherche d'autres diagnostiques comme l'ostéogénèse imparfaite car la maltraitance physique reste un diagnostic d’élimination.

## Conclusion

La maltraitance des enfants est un problème de plus en plus fréquent dans notre société. Excepté quelques cas cette maltraitance n'est pas prémédité, elle est souvent le résultat d'une colère ou d'une situation ponctuelle. Sur le plan orthopédique les associations lésionnelles causées par cette maltraitance chez des enfants en bas âge, prête à équivoque avec des diagnostics sérieux tels quel'ostéomyélite ou les lésions tumorales. Une meilleure connaissance des différents aspects du syndrome de Silverman permet de redresser le diagnostic. Concernant notre série, l’évolution clinique et une meilleure approche sociale nous ont permis de poser le diagnostic de maltraitance. Le médecin reste dépourvu de solutions face à ce problème, en effet après le volet médical un volet psycho-social doit être entrepris afin d’éviter la récidive, une meilleure coordination entre les différentes structures est requise.
